# Expression of glucocorticoid-receptor covaries with individual differences in visual lateralisation in zebrafish

**DOI:** 10.1007/s10071-025-01943-4

**Published:** 2025-03-13

**Authors:** Eleonora Rovegno, Elena Frigato, Luisa Dalla Valle, Cristiano Bertolucci, Tyrone Lucon-Xiccato

**Affiliations:** 1 Department of Life Sciences and Biotechnology, University of Ferrara, Ferrara, Italy; 2 Department of Biology, University of Padova, Padova, Italy

**Keywords:** Cognitive ecology, *Danio rerio*, Fish cognition, Individual differences, Laterality

## Abstract

Cerebral lateralisation, the differential cognitive processing in the two brain hemispheres, is variable among individuals in most vertebrates. Part of this variance has been attributed to plasticity in response to environmental stressors experienced by individuals and might be therefore mediated by the action of glucocorticoids (GCs). Accordingly, we tested the hypothesis that the GC pathway related to stress, which involved its cognate receptor GR, affects individuals’ lateralisation. First, we characterised the behavioural lateralisation phenotype of outbred wild-type zebrafish using three different tests: a motor test, a test involving a visual social stimulus (subject’s mirror image), and a test with a visual stimulus of negative valence (predator). Subsequently, we quantified the expression of the *gr* gene in the brain of the subjects, specifically in the telencephalon and mesencephalon of each hemisphere. Our zebrafish population exhibited individual variation but no population-level bias in behavioural lateralisation and *gr* expression across the two hemispheres. When we correlated the lateralisation patterns in the behavioural tests with *gr* expression, we observed that individuals with higher mesencephalic expression of *gr* in the right hemisphere were more inclined to process their mirror image using the right hemisphere. Additionally, individuals with higher *gr* expression in the telencephalon, showed reduced lateralisation in processing the predator stimulus. This study supports the hypothesis that GCs might affect some aspects of lateralisation, in particular those related to visual stimuli, thought the GC-Gr pathway and suggests that intraspecific variance in lateralisation could result from individual differences in *gr* expression.

## Introduction

In all the main groups of vertebrates, from teleost fish to mammals, specific cognitive functions tend to be processed primarily in one of the hemispheres (reviewed in Bisazza et al. 1998; Csermely and Regolin 2013; Vallortigara et al. 1999). This functional lateralisation is usually characterised by a large interindividual variation (e.g., Dadda et al. 2012). In the same population, some individuals perform a certain cognitive task preferentially with the right hemisphere, some with the left hemisphere, and others show no hemispheric preference. The evolutionary causes of this variability have been associated to trade-offs between costs and benefits of lateralisation (Vallortigara 2006). For instance, lateralisation enhances processing of multiple stimuli simultaneously (Dadda and Bisazza 2006), but determines visual and motor asymmetries preventing or slowing down responses to stimuli located on one side of the body (Dadda et al. 2009).

In contrast to the efforts in investigating the evolutionary causes of lateralisation variation, the underlying mechanisms are less known. A growing body of literature indicates that lateralisation varies in response to the environment experienced by the individuals (Dadda and Bisazza 2012; Lucon-Xiccato et al. 2020b; Rogers 1990). Most of the cases in which lateralisation plasticity has been observed are related to stressful conditions, such as presence of predators (Lucon-Xiccato et al. 2016, 2020a; Pellitteri-Rosa and Gazzola 2018), pollution (Besson et al. 2017; Domenici et al. 2012; Lucon-Xiccato et al. 2014; Merola et al. 2024), parasites (Roche et al. 2013), and maternal separation in mammals (Barnard 2016; Mundorf et al. 2020). Accordingly, it has been proposed that glucocorticoids (GCs), the main hormones involved in the stress responses of vertebrates, can be one of the mechanisms determining individuals’ lateralisation (Ocklenburg et al. 2016) and two studies provided some empirical support in damselfish and chickens (Ferrari et al. 2017; Henriksen et al. 2013). GCs act through both non-genomic mechanisms, such as interactions with membrane-bound receptors, and genomic pathways (Stahn and Buttgereit 2008). In the genomic pathway, glucocorticoid receptors (GRs) and mineralocorticoid receptors (MRs) are activated by binding to GCs in the cytosol. Upon activation, these receptors translocate to the nucleus and regulate gene transcription (Sacta et al. 2016). MRs, due to their higher affinity for GCs, are predominantly occupied at basal GC levels, while GRs are activated in response to stress-induced increases in GC levels (Sapolsky et al. 2000). Consequently, GRs are the primary candidates for linking stress and glucocorticoids to lateralisation.

In this study, we tested the hypothesis that individuals’ lateralisation is affected by GCs via activation of Gr, exploiting the zebrafish, *Danio rerio*. A considerable body of research on lateralisation plasticity has been conducted on fish (reviewed in Lucon-Xiccato and Bisazza 2024) and the zebrafish is an emerging model to understand GCs signalling, particularly useful, with respect to other teleost fish, due to the presence of a single copy of the *gr* gene coding for Gr (reviewed in Dinarello et al. 2020). We therefore focused on *gr* expression as an indicator for Gr abundance. In our experiment, we first scored lateralisation of individual zebrafish subjects from an outbred, wild-type population using the three most-used behavioural lateralisation tests for fish chosen to encompass as much as possible different aspects of lateralisation: the rotational preference test to assess motor lateralisation (Bisazza 1996; Dadda et al. 2010a, b, 2012; Izvekov et al. 2014); the mirror test to assess lateralised responses to a visual stimulus perceived as a conspecific (Dadda et al. 2010a, b; Lucon-Xiccato and Dadda 2017; Lucon-Xiccato et al. 2020c); and the detour test recording eye preference during the inspection of a potential predator (Dadda and Bisazza 2016; Dadda et al. 2010a, b; Lucon-Xiccato et al. 2020a). After completing the lateralisation phenotyping, we measured *gr* expression in the brain with a quantitative RT-qPCR approach. If individuals’ lateralisation is affected by GCs via activation of Gr, we expected to observe correlations between the levels of *gr* expression and the individuals’ scores in the behavioural lateralisation tests. However, due to the lack of previous studies on this relationship, we could not explicitly predict which aspects of lateralisation and gene expression would be implicated.

## Materials and methods

### Experimental fish

Adult zebrafish of an outbred, wild-type population kept in the fish facility at University of Ferrara were used in this study. The zebrafish population originated from approximately 100 zebrafish bought from a local shop in 2011. At the time of the study the population consisted of approximately 2000 individuals descendant from the original stock (approximately 25 generations). We kept the population relatively large to ensure low inbreeding and high diversity among individuals. The fish were housed in several 200-L and 50-L glass tanks (115 × 40 × 50 cm and 60 × 40 × 40 cm), in groups of approximately 20–40 individuals. The density of fish was kept low to ensure high welfare standards necessary for behavioural studies. These maintenance tanks were kept under standard laboratory conditions such as 14:10 h light:dark photoperiod and 27 ± 1 °C temperature. The water was constantly filtered with mechanical, biological, and chemical filters, to ensure optimal water parameters (pH 7 ± 0.2; conductivity 606.7 ± 60.18 µS/cm; nitrite below 0.1 mg/L; and nitrate below 50 mg/L). The fish were fed two/three times per day with live *Artemia salina* nauplii and dry food (Staple food Vipan, Sera, Heinsberg, Germany).

The population is maintained by routinely inducing spawning. This is done by inserting small apparatuses that simulated shallow water habitats in the maintenance tanks. Eggs obtained from spawning are collected in several petri dishes (10 cm Ø, h:1.5 cm) in a solution of E3 1 × (5 mM NaCl, 0.17 mM KCl, 0.33 mM CacCl_2_, 0.33 mM MgSO_4_) and Methylene blue (0.0016 g/l). Upon hatching, larvae are transferred into petri dishes with Fish Water solution 1 × (0.5 mM NaH_2_PO_4_*H_2_O, 0.5 mM Na_2_HPO_4_*H_2_O, 0.15 g Instant Ocean, 1 L deionized H_2_O). From the age of 6 dpf, larvae are transferred in 2-L tanks and feed with powdered food with zooplankton (18% krill) and phytoplankton (51% spirulina) (SERA Micron Nature) three times per day. Fish are then moved to the main housing tanks at approximately 1.5 months of age, where they are kept through adulthood. The fish from multiple spawning events are randomly assigned to different aquaria to reduce inbreeding. To further reduce inbreeding, once or twice per year we also added 100–200 new individuals (corresponding to approximately 5% of the laboratory population) from the shop. These individuals were used as breeders along with fish from the facility, and we ensured that offspring were not used as subjects before a couple of generations.

The subjects used in the study were zebrafish selected based on similar age (1.5 year) and size from the laboratory population. At this age, zebrafish reach large size in our facility, which was useful to improve accuracy during brain dissection. The subjects were exposed to the aforementioned housing conditions before the experiments. We selected groups of 5 subjects from 6 different maintenance tanks to reduce possibility of testing highly genetically related subjects. The initial sample size included 30 subjects, but one did not move in the first test and was excluded (final sample size *N* = 29). Due to the lack of studies linking lateralisation to receptor gene expression, this sample size was chosen based on earlier studies on learning (Gatto et al. 2024; Lucon-Xiccato et al. 2022b). Considering that the goal of the study was to investigate individual differences, this sample size corresponded to the number of biological replicates (*N* = 29). The subjects were collected from aquaria with mixed-sex (50:50 males:females ratio) fish to ensure testing fish of both sexes. The subjects underwent the three behavioural lateralisation tests sequentially (i.e., rotational preference, mirror, and detour test). This approach ensured that all the individuals have the same experience and the same eventual carry-over effects due to testing, thereby allowing to reduce confounds on our measures of individual differences (Bell 2013).

### Rotational preference test

**Fig. 1:**
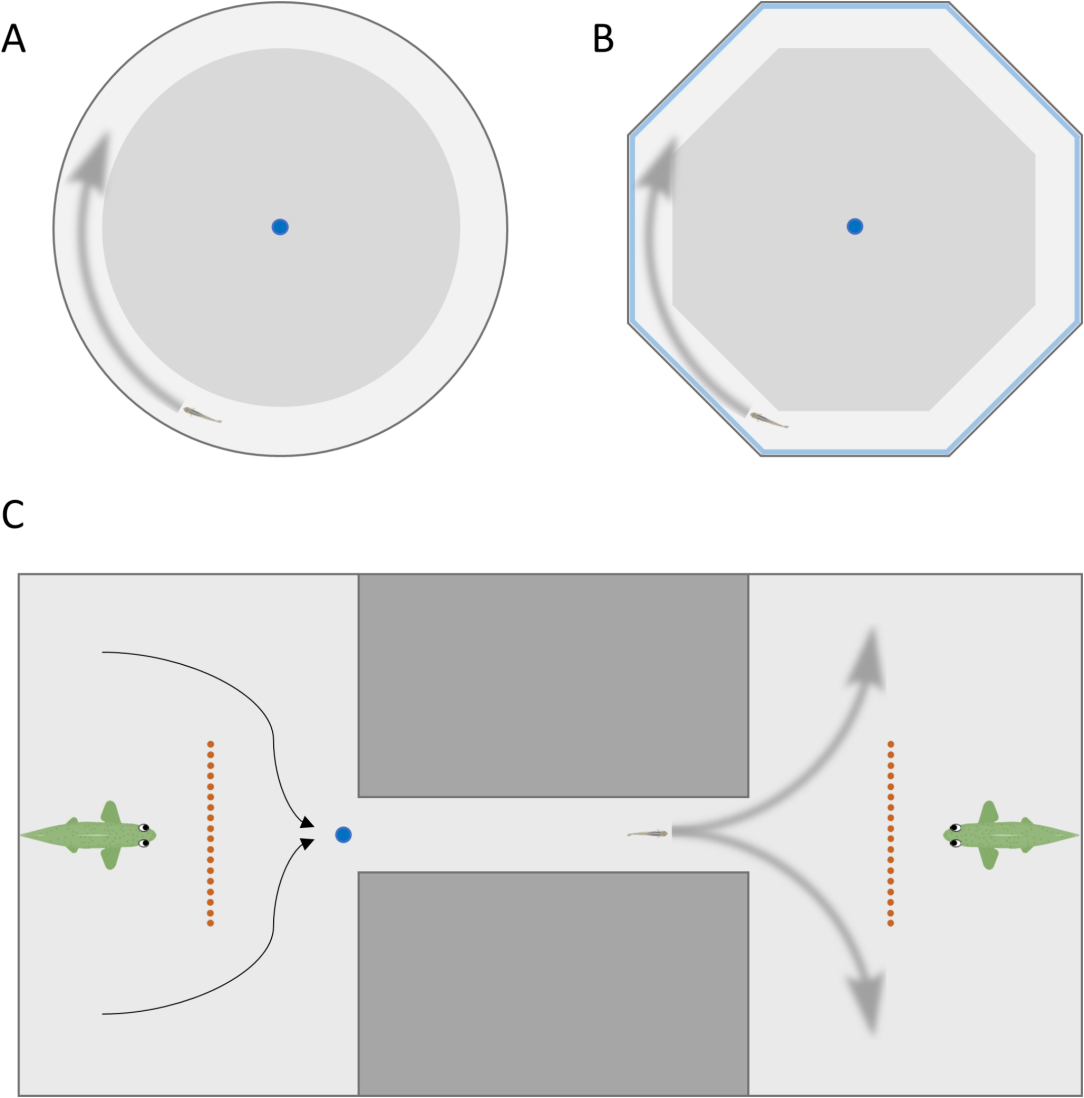
Schematic view from above of the experimental tanks of the (**A**) rotational preference test, the (**B**) mirror test, and the (**C**) detour test. Lighter areas in A and B represents the sectors used to score lateralisation. In A and B, blue dot indicates the starting position, blurred grey arrows represent an example of swimming direction. In C, blue dot represents the starting position, black arrows indicate the symmetrical slow movements of the nets used to direct the subject in the corridor, blurred grey arrow represent the two possible turns of the subject

To assess motor lateralisation in the absence of specific visual stimuli, we used a rotational preference test, which is often used in fish, including zebrafish (Bisazza 1996; Dadda et al. 2010a, b, 2012; Izvekov et al. 2014). The test exploited a circular arena, empty and with no visual stimuli, in which the fish were observed while spontaneously swimming (Fig. 1a). In this setting, fish often swim along the edges. This swimming behaviour along the edges determined a rotation of the fish in either clockwise or anticlockwise direction, which we used to estimate motor lateralisation.

To begin the test, one subject was randomly collected from the maintenance tanks and moved into a plastic jar (8 × 8 cm). After transporting the jar into the experimental room (approximately 30 s), it was gently emptied into the centre of the arena (40 cm diameter; water level 6 cm). The arena was made of white plastic and was uniformly illuminated from above by warm-white LED light strips (Superlight Technology Co. Ltd., Shenzhen, China). The subject was video-recorded using a camera placed on the ceiling (HDR-CX405, Sony Europe B.V., Weybridge, UK). The recording started from the moment in which the subject was released and lasted 30 min. The procedure was repeated for different individuals after changing the water in the apparatus.

The recordings were played back on a computer for the analyses of subjects’ behaviour. The analyses were conducted with the software Ciclic timer (1.3). The software consisted of a series of stopwatches that could be activated pressing the different keys of the computer keyboard. An experimenter selected predetermined keys to activate the stopwatches based on the position of the subject. One key was used to calculate the time spent by the subject in the centre of the apparatus, at more than 1 average body length (i.e., 4 cm) from the edges and another key was used for time spent in the edge sector motionless. Two other keys were used to obtain the temporal data necessary to score lateralisation: the time spent by the subject swimming in clockwise direction in the edge and the time spent by the subject swimming in anticlockwise direction in the edge. One fish that remained motionless throughout the entire test was excluded from the study, as it was potentially highly stressed or unwell. Following previous studies (Dadda et al. 2010a, b; Lucon-Xiccato et al. 2020c), we used the time spent swimming in the two directions to compute two lateralisation indices. The first index described relative lateralisation and was calculated for each subject as follows: (clockwise swimming time - anticlockwise swimming time) / (clockwise swimming time + anticlockwise swimming time). The relative lateralisation index could have values between − 1 and + 1, and indicated both the strength and the direction of lateralisation. The second index was the absolute lateralisation index, calculated as the absolute value of the relative lateralisation index. This second index described the strength of lateralisation of an individual irrespectively of the direction (values between 0, indicating low lateralisation level, and + 1 indicating high lateralisation level).

### Mirror test

The mirror test aimed to measure the lateralisation of visual processing of a stimulus with positive valence (social stimulus). This social stimulus consisted of the subject’s reflection on a mirror. The zebrafish are believed to perceive their mirror image as a conspecific (Pham et al. 2012; Qiu et al. 2021; Diakos et al. 2024). Following previous studies in fish (Dadda et al. 2010a, b; Lucon-Xiccato and Dadda 2017; Lucon-Xiccato et al. 2020c), we used an octagonal apparatus with eight mirrors in correspondence of the walls (side length 17 cm; mirror height 20 cm; water level 8 cm; Fig. 1b). In this setting, lateralised individuals tend to observe their mirror image with a specific eye. Due to the shape of the arena, the eye preference determined the subjects’ swimming direction. Zebrafish observing the stimulus with their left eye were more likely to swim in clockwise direction. Consequently, because visual information is processed by the opposite hemisphere, a preference for clockwise swimming suggested that the individual predominantly used its right hemisphere to process the visual stimulus.

Each subject was inserted into the centre of the mirror apparatus after collection from the arena of the previous test. Immediately after inserting the subject, its behaviour was video-recorded (HDR-CX405, Sony Europe B.V., Weybridge, UK) for 30 min. The video recordings were played back on a computer. An experimenter coded the behaviour of the fish with the Ciclic timer software as described in the previous test. The obtained data was the time spent swimming in clockwise and anticlockwise direction within 1 body length from the mirrors, which were used to compute the lateralisation indices, the time spent in the edge motionless, and the time spent in the centre of the arena.

### Detour test

The third lateralisation assay was the detour test, in which the subject is presented with a visual stimulus representing a potential predator (Dadda and Bisazza 2016; Dadda et al. 2010a, b; Lucon-Xiccato et al. 2020a). The test enabled us to determine which eye the fish preferred to use when observing the predator stimulus based on its turning behaviour. For example, a right turn when approaching the stimulus indicated that the fish was observing it with the left eye, meaning the information about the potential predator was processed in the right hemisphere.

The apparatus was based on the one used by Bisazza et al. (1997; Fig. 1c). It consisted of a rectangular tank (110 × 60 × 20 cm; water level: 8 cm), with two areas (30 × 60 cm) connected by a corridor (4 × 40 cm). In each of the two areas, a plastic brightly coloured predator-like fish (length: 12 cm) was placed behind a see-through barrier that blocked direct access to the stimulus. This predator-like stimulus was green with 3d eyes, its shape was symmetrical and resembled an Indian leaf fish, zebrafish natural predator (Bass and Gerlai 2008). Given that the subjects’ experience before the experiments was the laboratory environment, they were not familiar with the stimulus.

After being inserted in the apparatus, the subject was guided to enter the corridor for ten times using a pair of small nets (8 × 5 cm, brown net). The use of nets is typical of this procedure to ensure that the fish move between the compartments rapidly without familiarising with the stimulus (Reddon and Hurd 2009; Brown et al. 2007; Facchin et al. 1999; Bisazza et al. 1997). Each time the subject exited the corridor, it faced the barrier with the predator stimulus behind and had to turn either to the left or to the right. We considered a ‘turn’ when the subject exited the corridor and disappeared from the experimenter’s view, who was looking along the corridor from the behind the centre of the short side of the apparatus. A rightward turn would indicate that the subject inspected the predator stimulus with the left eye, processing it with the right hemisphere, and vice versa. The experimenter recorded the right and the left turns of the subjects across the ten trials to calculate the lateralisation indices as: (number of rightward turns - number of leftward turns) / (number of rightward turns + number of leftward turns). All the fish performed the 10 trials within 7.21 ± 3.39 (mean ± SD) minutes and were included in the sample of this experiment (*N* = 29).

### Gene expression analysis

In the gene expression analysis, we considered both the whole brain and the two hemispheres separately, because they could differ in gene expression (Lorenzi et al. 2019). We additionally analysed separately the expression in the two main brain areas in fish, the telencephalon and the mesencephalon. The 29 zebrafish that completed the three behavioural lateralisation tests were sacrificed within a short interval (i.e., 2 minutes) to quantify basal levels of *gr* expression of each individual. Fish were euthanized by MS-222 overdose and brain collected in RNAlater solution. Under dissection microscope, each brain was divided in 4 parts: right and left telencephalon and mesencephalon. Olfactory bulbs, diencephalon and cerebellum were discarded due to the difficulties in precisely dividing the left and the right part. We also decided not to include them as whole areas as pilot studies suggested that their dissection resulted in partial loss of the tissue. RNA was extracted using Trizol reagent following manufacturer’s instructions. After retrotranscription, we quantify via qPCR the expression of *gr* and the housekeeping gene *ef1a*. RNA amount and quality were analysed by BioSpec-nano (Shimadzu, Kyoto, Japan). DNaseI-treated RNA was used to perform cDNA synthesis using iScript cDNA Synthesis Kit (Bio-Rad Laboratories, Hercules, CA, USA). First-strand cDNA was PCR amplified with a CFX Connect Real-Time PCR Detection System (Bio-Rad Laboratories, Hercules, CA, USA) using SsoAdvanced ™ Universal SYBR ^®^ Green Supermix (Bio-Rad Laboratories, Hercules, CA, USA). After amplification, a melting curve analysis to confirm the specificity of the amplicon was performed. Gene-specific primers for *gr* (FOR: 5’-CAACACAATTACCTGTGTGCTG-3’; REV: 5’-CTTGACGTGCCTTTGACTTGC-3’; Dinarello et al. 2022) and *ef1a* (FOR: 5’-GACAAGAGAACCATCGAG-3’; REV: 5’-CCTCAAACTCACCGACAC-3’) were used. The efficiency of the *gr* primers was 99.3% (slope − 3.338). qPCR assays were performed in triplicate and the 2^–ΔΔCT^ method (where CT is the cycle number at which the signal reaches the threshold of detection) was used for expression quantification (Livak and Schmittgen 2001), using as reference sample for ΔΔCT calculation the sample with the highest Ct value, so the sample with the lowest gene expression. *gr* mRNA levels are showed in Fig. 3 in percentage as mean +/- SE for the different groups.

### Statistical analysis

Statistical analyses were conducted in R version 4.2.2. We used one sample t-tests to compare the lateralisation indices against zero. A relative lateralisation index different from zero would indicate the presence of an overall side bias in lateralisation (e.g., most of the individuals are lateralised in the same direction). An absolute lateralisation index greater than zero would indicate the presence of marked lateralisation, irrespective of the direction.

We subsequently looked for correlations between lateralisation indices of the three tests using the ‘cor.test’ function in R. Correlations were calculated with the Spearman method to avoid issues with the outliers. We always contrasted relative lateralisation indices and absolute lateralisation indices separately. Paired samples t-tests were used to investigate the expression levels of *gr* across the different brain areas investigated. We compared the expression levels between the following pairs of areas: overall right hemisphere versus the overall left hemisphere; right telencephalon versus left telencephalon; right mesencephalon versus left mesencephalon; overall telencephalon versus the overall mesencephalon; right telencephalon versus right mesencephalon; and left telencephalon versus left mesencephalon. The overall values of *gr* expression were computed as the sum of the separate parts.

Lastly, we used the ‘cor.test’ function to look for correlations using the Spearman method between the lateralisation indices obtained in the three behavioural tests and the pattern of *gr* expression. Due to the lack of previous studies and therefore absence of specific predictions, we tested various independent hypothesis. We initially correlated the relative lateralisation index and the absolute lateralisation index obtained in each test with the *gr* expression in the whole brain analysed and in its areas. Then, we correlated the relative lateralisation indices of the three tests with an analogous relative lateralisation index of *gr* expression computed as: (expression of *gr* in right hemisphere - expression of *gr* in left hemisphere) / (expression of *gr* in right + expression of *gr* in left hemisphere). The indices of asymmetric expression of *gr* were also computed and analysed separately for the telencephalon and the mesencephalon.

In the rotational test lateralisation analyses, we excluded 1 subject that spent less than 5% (3.83%) of the time swimming along the edge. This subject was considered an outlier based on the score of the other subjects (mean = 37.47%; median = 38.00%; range 13.50–64.61%). Based on the same threshold, we excluded 3 subjects with extremely low scores (scores: 0%, 0.06%, and 0.39%) from the mirror test. As the amplification in the qPCR failed for some (20/116) samples, the gene expression analyses were conducted using the available samples.

## Results

### Rotational preference test

**Fig. 2:**
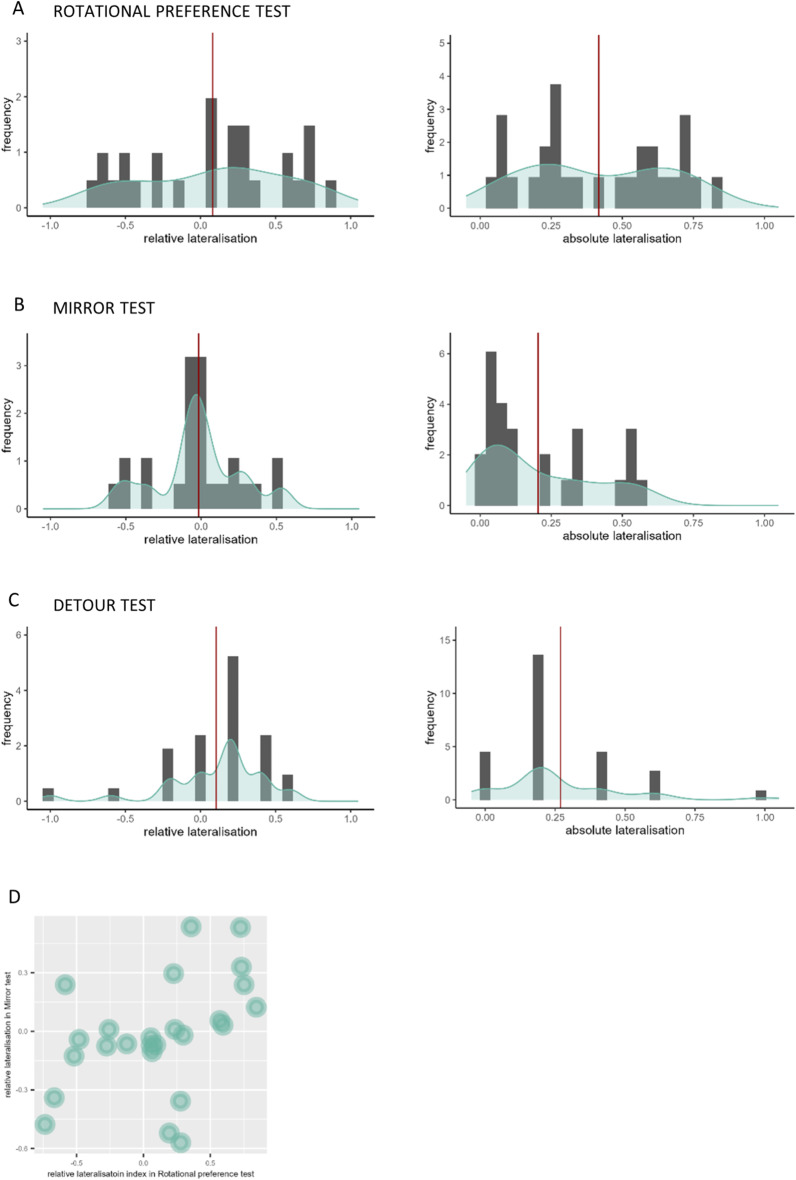
Frequency and density distribution of the relative and absolute lateralisation indices of the (**A**) rotational preference test, the (**B**) mirror test, and the (**C**) detour test; red vertical lines indicate the average lateralisation index, light blue line represents density. (**D**) Scatterplot of relative lateralisation index in the mirror test and the relative lateralisation index in rotational preference test

In the rotational preference test, subjects spent on average 10.64 ± 7.11% of testing time in the centre of the apparatus, 51.89 ± 16.63% of time along the edge without swimming, and 37.46 ± 13.83% (mean ± standard deviation) of time swimming along the edge of the apparatus. Our analyses indicated that approximately half of the time spent swimming along the edge was in clockwise direction (53.78 ± 24.13%). This resulted in a relative lateralisation index that was not significantly different from zero (0.081 ± 0.484; one sample t-test: t_27_ = 0.888, *P* = 0.382; Fig. 2a). The absolute lateralisation index was significantly greater than zero (0.417 ± 0.247; t_27_ = 8.946, *P* < 0.001).

### Mirror test

Subjects spent on average 15.46 ± 13.14% of testing time in the centre of the apparatus, 28.43 ± 16.77% of time along the mirror without swimming, and 56.11 ± 25.33% of time swimming along the mirrors. They spent 49.14 ± 14.18% of the swimming time close to the mirror swimming in clockwise direction, and therefore looking at the mirror image with their left eye. This resulted in a relative lateralisation index not significantly different from zero (-0.017 ± 0.284; one sample t-test: t_25_ = 0.310, *P* = 0.760; Fig. 2b). The absolute lateralisation index was significantly greater than zero (0.204 ± 0.194; t_25_ = 5.353, *P* < 0.001).

### Detour test

Data collected from the detour test indicated the subjects turned rightward in 55.17 ± 16.82% of trials. This resulted in a relative lateralisation index that was not significantly different than zero (0.103 ± 0.360; one sample t-test: t_28_ = 1.656, *P* = 0.109; Fig. 2c). The absolute lateralisation index was significantly greater than zero, indicating substantial strength in lateralisation in the detour test (0.269 ± 0.222; t_28_ = 6.519, *P* < 0.001).

### Correlation between lateralisation tests

The correlation analyses between the lateralisation tests indicated a significant relationship only between the relative lateralisation index in the rotational preference test and that in the mirror test (Spearman’s rank correlation test: *n* = 25, ρ = 0.532, *P* = 0.007; Fig. 2d). The relative lateralisation index of the detour test did not significantly correlate with those of the rotational preference test and the mirror test (*n* = 28, ρ = 0.007, *P* = 0.972; *n* = 26 ρ = 0.082, *P* = 0.691, respectively). The absolute lateralisation indices of the three tests did not significantly correlate (rotational preference test versus mirror test: *n* = 25, ρ = 0.321, *P* = 0.118; rotational preference test versus detour test: *n* = 28, ρ = -0.092, *P* = 0.641; mirror test versus detour test: *n* = 26, ρ = -0.139, *P* = 0.499).

### Gene expression

**Fig. 3:**
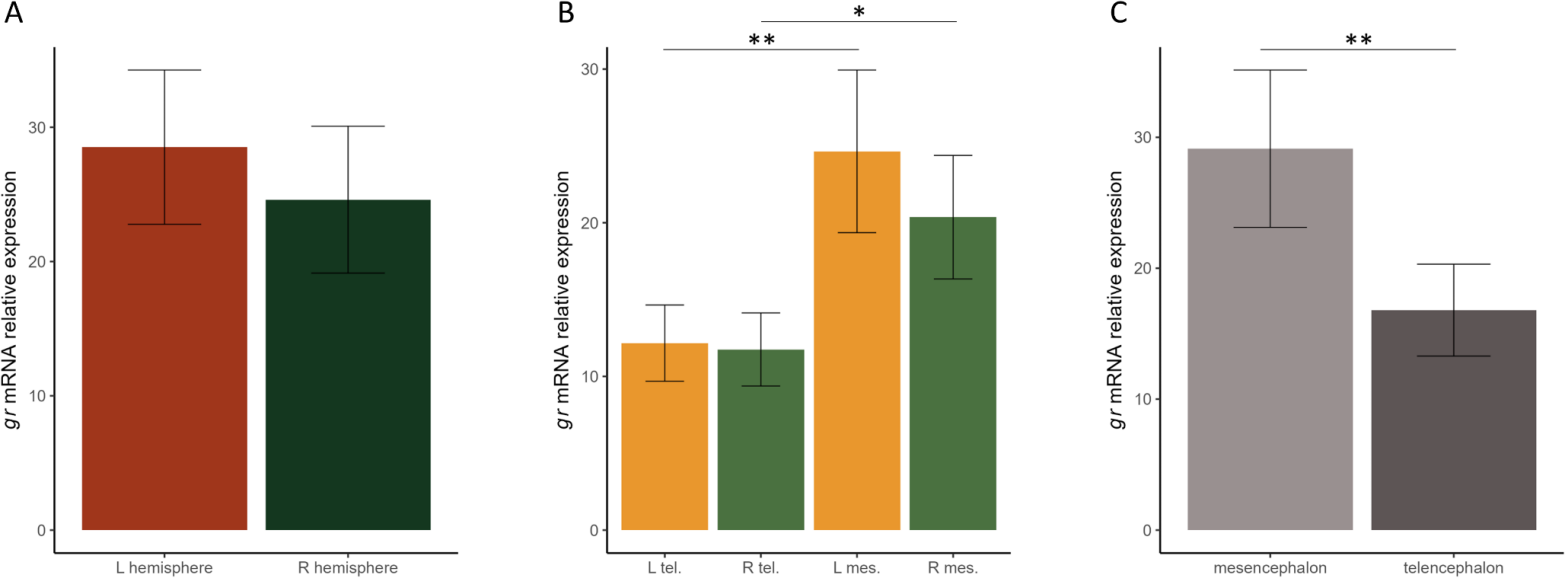
*gr* relative expression level in different brain areas (mean ± SE). Relative expression levels (100% is the maximum *gr* mRNA level detected) are plotted. Comparison between (**A**) left and right hemisphere, (**B**) telencephalon and mesencephalon, divided in R = ‘right’ and L = ‘left’, (**C**) mesencephalon and telencephalon

The comparison of *gr* expression levels across the different brain areas indicated no significant differences between the right and the left hemispheres overall (paired samples t-test: t_15_ = 0.452, *P* = 0.658; Fig. 3). An analysis considering separately the telencephalon (t_17_ = 0.213, *P* = 0.834; Fig. 3) and the mesencephalon (t_21_ = 1.192, *P* = 0.247; Fig. 3) confirmed the absence of asymmetric expression of *gr*.

Expression differences emerged when comparing the telencephalon and the mesencephalon within the same hemisphere: both in the right (t_17_ = 2.919, *P* = 0.010) and the left hemisphere (t_20_ = 3.738, *P* = 0.001), *gr* was more expressed in the mesencephalon (Fig. 3). Accordingly, a pooled analysis of the two hemispheres found a significant difference between telencephalon and mesencephalon (t_15_ = 3.528, *P* = 0.003; Fig. 3).

### Relationship between lateralisation and overall gene expression levels

**Fig. 4:**
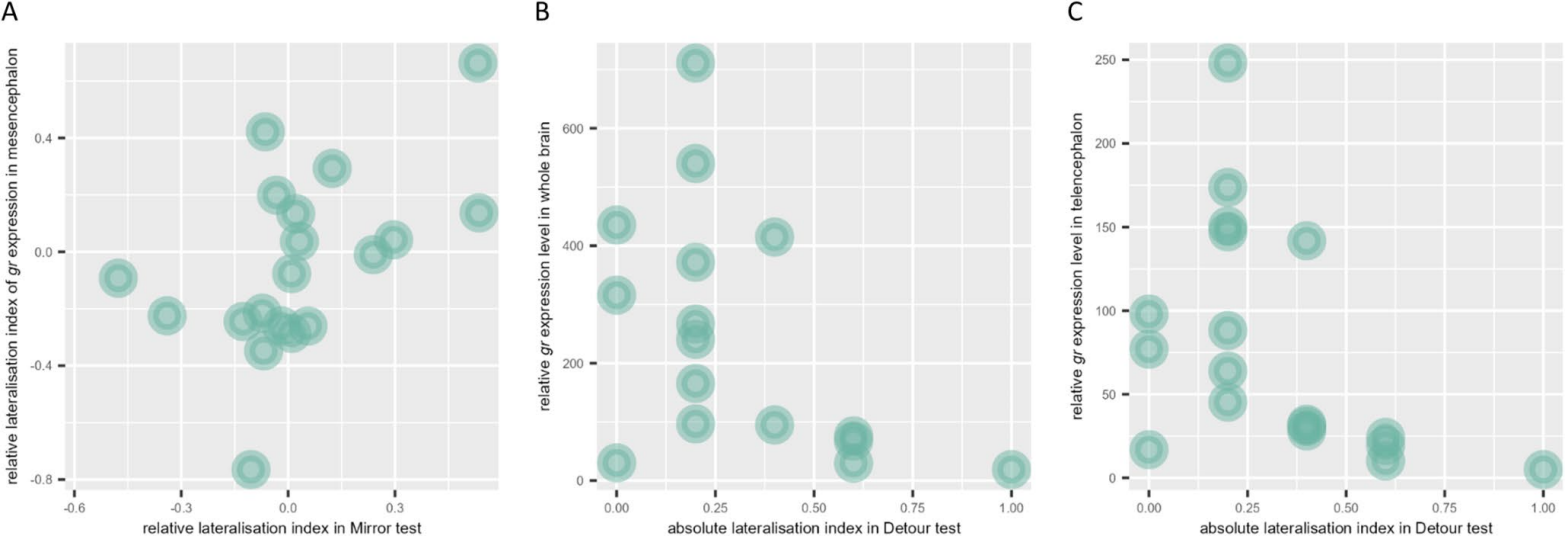
Scatterplot of the relationship between behavioural lateralisation and gr expression in the brain. (**A**) Relative lateralisation index in the mirror test versus relative lateralisation index of gr expression in the mesencephalon. (**B**) Absolute lateralisation index in the detour test versus relative gr expression level in the whole brain (telencephalon + mesencephalon); (**C**) Absolute lateralisation index in the detour test versus relative gr expression level in the telencephalon

In the rotational preference test, the relative lateralisation index and the absolute lateralisation index did not correlate with *gr* expression in the whole brain sample and in the two separate areas (Table 1). Similarly, in the mirror test, the relative lateralisation index and the absolute lateralisation index did not correlate with *gr* expression levels (Table 1). In the detour test, the relative lateralisation index did not correlate with *gr* expression (Table 1). The absolute lateralisation index in the detour test was instead significantly correlated with *gr* expression in the whole brain (Table 1; Fig. 4b) and in the telencephalon (Table 1; Fig. 4c), but not in the mesencephalon (Table 1).

**Table 1:** Spearman’s rank correlation tests between the lateralisation indices and the amount of Gr expression in the whole brain sample and its areas. Bold indicates significant correlations

Test	Lateralisation index	Whole brain	Telencephalon	Mesencephalon
Rotational	Relative	*n* = 15, ρ = 0.325, *P* = 0.237	*n* = 17, ρ = 0.380, *P* = 0.133	*n* = 21, ρ = 0.097, *P* = 0.674
Rotational	Absolute	*n* = 15, ρ = -0.229, *P* = 0.411	*n* = 17, ρ = -0.152, *P* = 0.559	*n* = 21, ρ = -0.339, *P* = 0.133
Mirror	Relative	*n* = 13, ρ = 0.429, *P* = 0.146	*n* = 15, ρ = 0.443, *P* = 0.100	*n* = 19, ρ = 0.125, *P* = 0.610
Mirror	Absolute	*n* = 13, ρ = − 0.181, *P* = 0.554	*n* = 15, ρ = -0.132, *P* = 0.639	*n* = 19, ρ = -0.228, *P* = 0.346
Detour	Relative	*n* = 16, ρ = 0.029, *P* = 0.914	*n* = 18, ρ = − 0.043, *P* = 0.866	*n* = 22, ρ = 0.237, *P* = 0.288
Detour	Absolute	*n* = 16,** ρ = − 0.533**, ***P*** **= 0.033**	*n* = 18,** ρ = − 0.558**, ***P*** **= 0.016**	*n* = 22, ρ = − 0.224, *P* = 0.317

### Relationship between lateralisation and asymmetric gene expression

The relative lateralisation index in the rotational test did not correlate with the relative lateralisation index in *gr* expression in the whole brain, in the telencephalon, and in the mesencephalon (Table 2). The relative lateralisation index in the mirror test correlated with the relative lateralisation index of *gr* expression in the mesencephalon (Table 2), but not in the whole brain and in the telencephalon (Table 2). In the detour test, the relative lateralisation index did not correlate with the relative lateralisation index of *gr* expression in the whole brain, in the telencephalon, and in the mesencephalon (Table 2).

**Table 2:** Spearman’s rank correlation tests between the lateralisation indices obtained in the behavioural tests and the lateralisation index of the Gr expression in the whole brain sample and its areas. Bold indicates significant correlations

Test	Lateralisation index of gr expression (whole brain)	Lateralisation index of gr expression (telencephalon)	Lateralisation index of gr expression (mesencephalon)
Rotational	*n* = 15, ρ = 0.282, *P* = 0.307	*n* = 17, ρ = -0.054, *P* = 0.839	*n* = 21, ρ = 0.283, *P* = 0.213
Mirror	*n* = 13, ρ = 0.396, *P* = 0.182	*n* = 15, ρ = − 0.011, *P* = 0.975	***n*** **= 19, ρ = 0.505**, ***P*** **= 0.029**
Detour	*n* = 16, ρ = − 0.156, *P* = 0.565	*n* = 18, ρ = 0.209, *P* = 0.405	*n* = 22, ρ = − 0.173, *P* = 0.442

## Discussion

Lateralisation in animal species is characterised by significant individual differences, which appear to be linked to the stress experienced by the individuals (reviewed in Lucon-Xiccato and Bisazza 2024). In this study, we explored the pathway involved in this relationship by correlating individuals’ lateralisation with the level of expression of *gr*, a transcript coding for a steroid receptor involved in responses to stress. Our experimental results indicate that *gr* expression influences some aspects of visual lateralisation, but not motor lateralisation, in zebrafish.

Our subjects exhibited individual differences in behavioural lateralisation, but there was no population-level alignment in the direction of lateralisation, in line with an earlier study with this laboratory stock of zebrafish (Lucon-Xiccato et al. 2020c). There was a significant positive correlation between the lateralisation scores in the rotational and in the mirror test, which could be attributed to either the covariation between the asymmetry of different cognitive functions or the involvement of a single mechanism (e.g., motor asymmetry) in the two tests. The expression of *gr* was greater in the mesencephalon, consistent with findings from an earlier study (Natsaridis et al. 2023). There was no evidence of population-level asymmetric *gr* expression between the two hemispheres, which can be related to the fact that our study sample did not show population-levels in the behavioural aspects of lateralisation. However, when we focused on the interindividual variation, we found that individual zebrafish with higher expression of *gr* in the right mesencephalon were more likely to process their mirror image with the right hemisphere in the mirror test. Moreover, individuals with higher *gr* expression, especially in the telencephalon, displayed reduced lateralisation in processing the predator stimulus.

These findings support the hypothesis formulated in other vertebrates that individual differences in lateralisation might be related to GRs, at least in the case of the response to visual social and predator stimuli. This suggests that the hormones activating the GR receptors, e.g., the corticosteroids, might affect lateralisation, although the exact modulation and the pathway involved were not addressed by the present study. Certainly, it will be important to perform studies similar to the present ones in other vertebrate groups to ensure that the effect we found is not limited to fish. Considering that the GCs pathways and lateralisation display some degree of homology across vertebrates (Rogers et al. 2013; Stolte et al. 2006; Charmandari et al. 2005; Vallortigara et al. 1999), a relationship between GR and lateralisation might be present also in other groups.

Our results further suggest that the effect of Gr on lateralisation in zebrafish is complex, depending on both the total expression of *gr*, *gr* expression asymmetries, and the specific task or type of lateralisation involved. Although there have been attempts to investigate the functions of brain regions in fish (Calvo and Schluessel 2021), achieving a full understanding of the neural substrates involved in the observed effects of *gr* on lateralisation remains challenging. As most of the neural substrates for social interaction identified in fish are located in the telencephalon (Ausas et al. 2019; Dunlap et al. 2021), the role of mesencephalic *gr* in the mirror test could be attributed to the inclusion of the optic tectum in this area. The optic tectum is part of the sensory pathways involved in the visual recognition of conspecifics and biological motion (O’Connell and Hofmann 2011; Kappel et al. 2022; Kelly 2002). This might explain why the significant covariations between lateralisation and *gr* expression were found in the two tests with a visual stimulus (i.e., the mirror test and the detour test). The effect of telencephalic *gr* in the detour task, which involves a predator visual stimulus, is likely due to the activation of various circuits in this area that are involved in threat processing and anti-predator responses (do Carmo Silva et al. 2018; Lal et al. 2018). Interestingly, the level of *gr* expression was apparently not involved in motor lateralisation as assessed with the rotational test. This is notable because Gr has also been reported in mesencephalic regions involved in motor control (Natsaridis et al. 2023). It is possible that in the absence of biologically relevant visual stimuli, as in the rotational preference test setting, other factors, such as locomotor routine and morphological biases, might be the main drivers in determining individual differences in behavioural lateralisation. It is worth noting that this study was the first attempting to connect lateralisation variation to gene expression, at least in zebrafish. This has led us to select the experimental design and its details such as the sample size based on studies conducted on other cognitive traits. Although we tried to be conservative, for instance doubling the sample size used in other studies (e.g., Gatto et al. 2024; Lucon-Xiccato et al. 2022b), we cannot exclude the presence of more covariations between lateralisation and *gr* levels that our study failed to detect.

A research question arising from our study concerns the mechanisms determining the variability in *gr* expression in fish brains. The *gr* expression is characterised by substantial plasticity due to the level of stress experienced by an individual (De Kloet et al. 1986). Moreover, there is plasticity in the actions of GR (Jimeno and Zimmer 2022). However, research suggests that heritable differences in *gr* expression patterns might also exist. Johansen and colleagues (2011) artificially selected lines of rainbow trout with different stress responses, finding that these processes also determined differences in *gr* expression in the lines, although only in the cerebellum. Therefore, the amount, and possibly the distribution, of *gr* in the fish brain might be controlled by both genetic and experiential factors, ultimately determining individual differences in lateralisation.

Future work should evaluate other aspects of the effect of Gr on lateralisation in fish, as well as studying the homologies receptors present in other vertebrates. Studies in other fish species have begun to report within-individual variance in lateralisation across different times of the day (Ferrari et al. 2017) and seasons (Lucon-Xiccato et al. 2022a). Given that in other vertebrates GR expression, turnover, and presence vary according to these cyclical conditions (Charmandari et al. 2011; Herman et al. 1993), this receptor likely plays a role in within-individual plasticity in lateralisation. It is also worth considering that GR is probably only one piece of the puzzle, and additional factors may contribute to the hormonal control of lateralisation variance. Among the most interesting candidates is the mineralocorticoid receptor (MR), which is also activated by these hormones even in the absence of stress. Furthermore, various other steroid hormones, including sex steroids, could be involved (Beking et al. 2017; Pfannkuche et al. 2009). As a support, several studies have reported sex differences in lateralisation in fish and other vertebrates (Hirnstein et al. 2019; Ocklenburg et al. 2016; Reddon and Hurd 2009; Alonso et al. 1991). All these aspects should be considered to gain a deeper understanding of individualities in lateralisation.

## Data Availability

Data will be provided by the corresponding author upon request.
