# Correction: Expression of glucocorticoid-receptor covaries with individual differences in visual lateralisation in zebrafish

**DOI:** 10.1007/s10071-025-01987-6

**Published:** 2025-07-26

**Authors:** Eleonora Rovegno, Elena Frigato, Luisa Dalla Valle, Cristiano Bertolucci, Tyrone Lucon-Xiccato

**Affiliations:** 1 Department of Life Sciences and Biotechnology, University of Ferrara, Ferrara, Italy; 2 Department of Biology, University of Padova, Padova, Italy

**Correction: Animal Cognition (2025) 28:21**

10.1007/s10071-025-01943-4

In this article the ‘Conflict of Interest’ statement was missing and should have been “Editor Tyrone Lucon-Xiccato is a guest editor for the journal and has not taken part in the manuscript’s peer review process”. The original article has been corrected.

